# Analytic validation of NeXT Dx™, a comprehensive genomic profiling assay

**DOI:** 10.18632/oncotarget.28490

**Published:** 2023-08-30

**Authors:** Juan-Sebastian Saldivar, Jason Harris, Erin Ayash, Manqing Hong, Prateek Tandon, Saloni Sinha, Patricia Miranda Hebron, Erin E. Houghton, Kaleigh Thorne, Laurie J. Goodman, Conan Li, Twinkal R. Marfatia, Joshua Anderson, Massimo Morra, John Lyle, Gabor Bartha, Richard Chen

**Affiliations:** ^1^Personalis, Inc., Fremont, CA 94555, USA; ^2^These authors contributed equally to this work

**Keywords:** comprehensive genomic profiling, whole exome sequencing, whole transcriptome sequencing, tumor-normal, precision medicine

## Abstract

We describe the analytic validation of NeXT Dx, a comprehensive genomic profiling assay to aid therapy and clinical trial selection for patients diagnosed with solid tumor cancers. Proprietary methods were utilized to perform whole exome and whole transcriptome sequencing for detection of single nucleotide variants (SNVs), insertions/deletions (indels), copy number alterations (CNAs), and gene fusions, and determination of tumor mutation burden and microsatellite instability. Variant calling is enhanced by sequencing a patient-specific normal sample from, for example, a blood specimen. This provides highly accurate somatic variant calls as well as the incidental reporting of pathogenic and likely pathogenic germline alterations. Fusion detection via RNA sequencing provides more extensive and accurate fusion calling compared to DNA-based tests. NeXT Dx features the proprietary Accuracy and Content Enhanced technology, developed to optimize sequencing and provide more uniform coverage across the exome. The exome was validated at a median sequencing depth of >500x. While variants from 401 cancer-associated genes are currently reported from the assay, the exome/transcriptome assay is broadly validated to enable reporting of additional variants as they become clinically relevant. NeXT Dx demonstrated analytic sensitivities as follows: SNVs (99.4%), indels (98.2%), CNAs (98.0%), and fusions (95.8%). The overall analytic specificity was >99.0%.

## INTRODUCTION

In 2023, it is estimated that physicians will diagnose 1,958,310 new cancer cases and 609,820 cancer deaths will occur in the United States [1]. Cancer is a leading cause of death in the U.S., and the prevalence has been expected to continue to increase over time. Investment in precision medicine through molecular testing to match specific tumor biomarkers with appropriate therapies could ease the high clinical burden and costs associated with cancer [2]. Precision medicine in oncology has been shown to lengthen survival, enhance patient quality of life, and improve economic outcomes [3, 4]. Unfortunately, there are multiple logistical challenges presented by genomic testing, especially in community-based treatment settings, and not all patients eligible for targeted therapy are receiving genomic tests that could result in a matched therapeutic [5]. Additionally, in non-small cell lung cancer (NSCLC), comprehensive testing is widely underutilized despite a growing number of clinically actionable alterations, and molecular testing in earlier stage disease has not been required even though evidence supports the use of targeted therapy in resectable disease [6–8].

Next generation sequencing (NGS) is an advanced technology used to identify driver mutations of cancer growth, including SNVs, insertions, deletions, copy number alterations, fusions, TMB and MSI. NGS-based cancer testing has become an invaluable tool to guide treatment decisions as well as determine eligibility for clinical trials, particularly in advanced cancer patients that failed prior therapy and/or have few therapeutic options [9]. Presently, most commercial NGS platforms lack scalability for future approved targets since they are limited to smaller gene panels that do not comprehensively cover potential future clinically relevant alterations [10, 11]. Furthermore, many commercially available tests are focused on recurrent hotspots and may not address all drug resistance mechanisms [12, 13]. More comprehensive assays with adaptability to future developments are needed to address the problem of sequential testing for specific or limited numbers of genes, which can require extended amounts of time, often exhausting precious tumor tissue samples as well as delaying or underdiagnosing patients with cancers amenable to treatment [14–17]. This is especially problematic in the community oncology setting where adoption of comprehensive genomic profiling for advanced cancer patients remains a challenge [5].

Many current genomic profiling tests utilize a tumor-only analysis approach which may be subject to a high false positive somatic mutation rate. This is in part because corrections for false somatic mutations utilize pools of healthy donors over-represented by individuals of European descent in public single nucleotide polymorphism (SNP) databases, and can therefore result in less-effective corrections for non-European patients [18]. Tumor specific alterations can be detected with greater accuracy using matched germline sequence subtraction, which results in identification of patient-specific somatic mutations regardless of ancestry [19]. For these reasons, in 2017, the Association for Molecular Pathology (AMP), American Society of Clinical Oncology (ASCO) and College of American Pathologists (CAP) jointly recommended tumor-normal testing: “Concurrent analysis of a paired germline sample is desirable because it clarifies interpretation” [20].

Inaccurate germline correction also affects the calculation of TMB, an important emerging biomarker for cancer immunotherapy. TMB has been shown to be overestimated using tumor-only sequencing panels versus those with germline subtraction [21]. In one study, tumor-only testing was found to provide false TMB-high calls (defined as >10 mut/Mb) in 21–44% of cases, with patients of African ancestry having the highest false positive rate [22]. Studies have also indicated that DNA-based assays for gene fusion detection can miss certain fusions because of the need to sequence large intronic regions, which is highly inefficient using DNA sequencing [23]. Functional gene fusions can be determined most accurately across a broad set of target genes from RNA sequencing which detects gene fusions that are expressed. For this reason, ASCO, through a Provisional Clinical Opinion, recently recommended that gene fusions be determined using RNA sequencing [24].

Tumor DNA can be efficiently profiled in a single assay by whole exome sequencing (WES) to evaluate for molecular biomarkers. Nevertheless, there is variation of coverage and sensitivity of the portions of the genome relevant to medical treatments in conventional exome-capture and currently available WES platforms, including in areas of known disease-associated loci in the genome [25].

Commercially available WES platforms can have uneven coverage across difficult-to-sequence gene regions and clinically important variants can be missed. Thus, a more comprehensive test that covers all clinically relevant alterations and is adaptable to changes in the future is essential in oncology.

To overcome these obstacles, we developed and analytically validated the NeXT Dx tumor-normal whole exome and whole transcriptome sequencing assay (“NeXT Dx”). NeXT Dx is a comprehensive genomic profiling test for patients diagnosed with a new, progressive, or recurrent solid tumor malignancy, including sarcoma. The NeXT Dx test utilizes NGS of the entire >20,000-gene exome to accurately detect somatic variants (SNVs, indels, CNAs, and TMB), from DNA. MSI is determined from 117 loci in the exome. RNA fusions are detected from sequencing the transcriptome at 200 million reads. Additionally, pathogenic and likely pathogenic germline variants in 59 cancer-related genes are reported as incidental findings from the patient’s matched normal sample. NeXT Dx was developed based on the proprietary ACE augmented exome technology which offers increased sensitivity compared to current genomic profiling platforms due to specific expansion of coverage into translated and untranslated gene regions, including difficult-to-sequence GC-rich regions; this provides improved coverage in genomic regions that are clinically relevant.

NeXT Dx is a laboratory developed test (LDT), a single-site assay performed at a Clinical Laboratory Improvement Amendments (CLIA)/College of American Pathologists (CAP)-certified clinical genomics testing laboratory, the Personalis Clinical Laboratory (Personalis, Inc), located in Fremont, California. The NeXT Dx clinical report currently provides the ordering clinician with information from 401 cancer-related genes on clinically relevant mutations, as well as related drug response associations and a curated list of clinical trials that may be applicable to the patient.

## RESULTS

The performance of NeXT Dx was analytically validated by comparison to an orthogonal clinical reference method, the TruSight Oncology 500 (TSO500) platform (Illumina, San Diego), operated in an independent CLIA-certified clinical laboratory. The studies were performed on formalin fixed paraffin embedded (FFPE) and fresh frozen tumor tissue with matched normal specimens from adjacent normal tissue or frozen buffy coat samples. Supplemental fusion samples including a NRG1 positive cell line and a SeraCare construct were included to ensure fusion detection for rare events. A summary of specimens and tumor types utilized in the validation study are shown in Table 1A and the number of samples used to validate each variant type is provided in Table 1B.

**Table 1A:** Tumor sample types used in the analytical validation

Tumor type	Total	Small variants	CNA	MSI	TMB	Gene fusions
Astrocytoma	2	0	0	0	0	2
Bladder	22	21	21	20	21	19
Breast	26	13	23	13	13	16
Cervical	12	11	2	11	11	10
Colorectal	36	28	26	28	28	30
Gastric	3	3	3	3	3	3
Head & Neck	20	19	16	19	19	14
Liver	23	22	21	22	22	17
Melanoma	11	6	6	6	6	9
NSCLC	25	14	14	14	14	22
Ovarian	18	14	12	14	14	18
Pancreatic	17	16	5	16	16	16
Prostate	15	15	13	15	15	14
Renal cell carcinoma	18	17	17	15	17	15
Thyroid	20	4	4	4	4	18
Uterine	20	15	15	12	15	20
Cell lines or reference standard	2	0	0	0	0	2

**Table 1B:** Samples and variant types utilized in the analytic validation

Variants validated	Validation samples	Source of validation	Number of variants for validation
SNVs	218 clinical samples	Tumor DNA	2,986 SNV events
Indels	218 clinical samples	Tumor DNA	674 Indel events
Copy number alterations	198 clinical samples	Tumor DNA	100 CNA events
Gene fusions	244 clinical samples, 1 cell line, 1 SeraCare reference standard	Tumor RNA	121 fusion events
MSI	212 clinical samples	Tumor DNA	117 loci/sample
TMB	218 clinical samples	Tumor DNA	Exome-wide; Reported in mutations/Mb

### Assay performance quality metrics

Assay Quality Metrics used in the validation were established in advance of the study to encompass pre-analytical, analytical, and post-analytical processes. Specimens were analyzed by a pathologist for tumor content and samples that had tumor content ≥20% were included in the validation. The RNA input range was 50–200 ng based on a ≥30% DV200 value and the total sequencing reads were >200 million. Tumor DNA input ranged from 150–750 ng with a corresponding quality ratio of A260/230 of 1.7 to 2.9. The DNA sequencing depth was a median of >500× across the whole exome, with a 247-gene subset of cancer-related genes sequenced to a depth of >1500×. DNA from the normal specimen was sequenced at a median depth of 150×. The core quality metrics used in the validation are detailed in Table 2.

**Table 2:** Assay performance quality metrics

Metric	Details
RNA Quantity	50–200 ng input
RNA Quality	≥30% DV200
DNA Quantity	150–750 ng input
DNA Quality	A260/230 1.7–2.9
Tumor Content	≥20% input
Library Quantification (bp)	195–350 bp
Onboard Q30	≥75%
PhiX	~0.2–1%
Depth of coverage	Median 500×, Boosted region >1500×, Normal DNA 150×
Total reads (RNA)	200 million

### Overall performance

The analytical performance of NeXT Dx was determined by evaluating a variety of analytic parameters and determining accuracy, sensitivity, and specificity of all the variant types in the assay by comparing results with an established reference method performed in a CLIA-certified clinical laboratory. The overall performance of the assay is summarized in Table 3.

**Table 3:** Overall performance of NeXT Dx

	Variant	Specification
Analytic Sensitivity	Single Nucleotide Variants, AF≥5%	99.4% (CI 99.0–99.6%)
Analytic Sensitivity	Insertions/Deletions, AF≥5%	98.2% (CI 96.9–99.1%)
Analytic Sensitivity	Copy Number Alterations	98.0% (CI 93.0–99.8%)
Analytic Sensitivity	Gene Fusions	95.8% (CI 90.5–98.6%)
Analytic Specificity (PPV)	Specificity	>99%
MSI	117 Gene Loci	>99.1% Concordance
TMB	Exome Wide	0.985 Pearson correlation coefficient

Table 4 describes the cancer-related genes reported by NeXT Dx. Asterisks point out genes that may be reported as pathogenic or likely pathogenic incidental germline variants. The 284 fusion genes are indicated by daggers (†).

**Table 4:** NeXT Dx cancer-related genes

*ABCB1*^†^	*CAMTA1*^†^	*CSF1R*^†^	*EWSR1*^†^	*GATA1*^†^	*LIG4*	*MUTYH*^†^	*PDGFA*	*PTPN11*^†^	SDHA^*^	*TMPRSS2*^†^
*ABL1*^†^	*CBFB*^†^	*CSF3R*^†^	*EXO1*	GATA2^*†^	*LRP1B*	*MYC*^†^	*PDGFB*^†^	*PVRL4*^†^	SDHAF2^*^	*TNFRSF4*^†^
*AKAP9*^†^	*CBL*^†^	*CTAG2*^†^	*EZH2*^†^	*GEN1*	*MAGEA3*^†^	*MYCL*	PDGFRA^*†^	*RAD21*^†^	SDHB^*†^	*TNFRSF8*^†^
*AKT1*^†^	*CCNA1*	*CTDNEP1*	*EZHIP*^†^	*GLI2*^†^	*MAGEA4*^†^	*MYCN*^†^	*PDGFRB*^†^	RAD50^*†^	SDHC^*†^	*TNFRSF10B*
*AKT2*^†^	*CCNA2*	*CTLA4*^†^	*FAM175A*	*GNA11*^†^	*MAML1*	*MYD88*^†^	*PGR*^†^	*RAD51*^†^	SDHD^*†^	TP53^*†^
*AKT3*^†^	*CCNB1*	*CTNNA1*	*FAN1*	*GNAQ*^†^	*MAP2K1*^†^	*MYH11*^†^	*PHF1*^†^	*RAD51B*^†^	*SETBP1*^†^	TSC1^*†^
ALK^*†^	*CCNB2*	*CTNNA2*	*FANCA*^†^	*GNAS*^†^	*MAP2K2*^†^	*MYOD1*^†^	PIK3CA^*†^	RAD51C^*†^	*SETD2*	TSC2^*†^
APC^*†^	*CCNB3*^†^	*CTNNA3*	*FANCB*^†^	*GPNMB*^†^	*MAP2K4*^†^	*NAB2*^†^	*PIK3CB*^†^	RAD51D^*†^	*SF3B1*^†^	*TYRO3*
*APOBEC3B*	*CCND1*^†^	*CTNNB1*^†^	*FANCC*^†^	*H3F3A*	*MAP3K1*^†^	*NBN*	*PIK3CD*^†^	*RAD52*	*SHFM1*	*U2AF1*^†^
*AR*^†^	*CCND2*^†^	*CUX1*^†^	*FANCD2*^†^	*HDAC1*	*MAPK1*^†^	*NCSTN*	*PIK3CG*^†^	*RAD54B*	*SHH*^†^	*USH2A*
*ARAF*^†^	*CCND3*^†^	*DDR2*^†^	*FANCE*^†^	*HDAC2*	*MAPK11*	NF1^*†^	*PIK3R1*^†^	*RAD54L*	*SLX4*^†^	*VEGFA*^†^
*AREG*^†^	*CCNE1*^†^	*DDX3X*	*FANCF*^†^	*HEY1*^†^	*MAPK3*	NF2^*†^	*PIK3R2*	*RAF1*^†^	SMAD4^*†^	*VEGFB*^†^
*ARID1A*^†^	*CCNE2*	*DEK*^†^	*FANCG*^†^	*HNF1A*^†^	MAX^*^	*NFE2L2*^†^	*PML*^†^	*RARA*^†^	*SMARCA4*^†^	*VEGFC*
*ARID1B*	*CD274*^†^	*DKK1*^†^	*FANCI*^†^	*HRAS*^†^	*MBTD1*^†^	*NKX2-1*^†^	*PMS1*	RB1^*†^	*SMARCB1*^†^	*VGLL2*^†^
*ARID2*	*CD276*^†^	*DLL3*^†^	*FANCL*^†^	*HSP90AA1*^†^	*MCL1*^†^	*NOTCH1*^†^	PMS2^*†^	*RBBP8*	*SMC1A*^†^	VHL^*†^
*ASXL1*^†^	*CD40*^†^	*DLL4*	*FANCM*^†^	*IDH1*^†^	*MCPH1*	*NOTCH2*^†^	POLD1^*^	*RBM15*^†^	*SMC3*^†^	*WEE1*^†^
ATM^*†^	CDH1^*†^	*DNMT3A*^†^	*FBXW7*^†^	*IDH2*^†^	*MDC1*	*NOTCH3*^†^	*POLD2*	*RECQL4*	*SMO*^†^	*WRN*
*ATR*^†^	*CDH3*^†^	*DOT1L*	*FCER2*^†^	*IGF1R*^†^	*MDM2*^†^	*NOTCH4*	POLE^*†^	*RELA*^†^	*SRC*^†^	WT1^*†^
*ATRX*^†^	*CDK1*	*EED*	*FGF2*^†^	*IKBKE*	*MDM4*^†^	*NPAP1*	*POLQ*	RET^*†^	*SRSF2*^†^	*WWTR1*^†^
*AURKA*^†^	*CDK2*	EGFR^*†^	*FGF4*	*IKZF1*^†^	*MECOM*^†^	*NPM1*^†^	*PPM1D*	*RFC1*	*SS18*^†^	*XPO1*^†^
*AXL*^†^	CDK4^*†^	*EIF1AX*	*FGF19*^†^	*IL2RA*^†^	MEN1^*†^	*NR4A3*^†^	*PPP2R1A*	*RFC2*	*SSBP1*	*XRCC1*^†^
BAP1^*†^	*CDK6*^†^	*EML4*^†^	*FGFR1*^†^	*JAG1*	*MERTK*	*NRAS*^†^	*PPP2R2A*	*RFC3*	*STAG2*^†^	*XRCC2*
BARD1^*^	*CDK9*^†^	*EP300*^†^	*FGFR2*^†^	*JAK1*^†^	MET^*†^	*NRG1*^†^	*PRAME*^†^	*RFC4*	*STAT3*^†^	*XRCC3*
*BCL2*^†^	*CDK12*	*EPCAM*^†^	*FGFR3*^†^	*JAK2*^†^	*MGAM*	*NTRK1*^†^	*PRKACA*^†^	*RFC5*	*STAT5B*^†^	*XRCC4*
*BCL6*^†^	*CDKN1A*^†^	*EPHA2*	*FGFR4*^†^	*JAK3*^†^	*MKL1*^†^	*NTRK2*^†^	*PRKCA*^†^	*RHEB*	*STAT6*^†^	*XRCC5*
*BCOR*^†^	CDKN1B^*†^	*ERBB2*^†^	FH^*†^	*KDM5C*	MLH1^*†^	*NTRK3*^†^	*PRKCB*^†^	*RICTOR*^†^	STK11^*†^	*XRCC6*
*BCORL1*^†^	CDKN2A^*†^	*ERBB3*^†^	*FIGF*	*KDM6A*^†^	*MLH3*	*NUP214*^†^	*PRKCD*^†^	*ROS1*^†^	SUFU^*^	*YAP1*^†^
*BCR*^†^	*CDKN2B*^†^	*ERBB4*^†^	FLCN^*†^	*KDR*^†^	*MLLT3*^†^	*NUTM2A*^†^	*PRKCE*^†^	*RPA1*	*SULT1A1*^†^	*YES1*^†^
*BLM*	*CDKN2C*	*ERCC1*	*FLT1*^†^	*KEAP1*	*MPL*^†^	*OTX2*	*PRKCG*^†^	*RPA2*	*SUZ12*^†^	*YWHAE*^†^
*BRAF*^†^	CEBPA^*†^	*ERCC2*	*FLT3*^†^	KIT^*†^	MRE11A^*†^	PALB2^*†^	*PRKCI*^†^	*RPA3*	*SYK*^†^	*ZMYM3*
BRCA1^*†^	*CHEK1*^†^	*ERCC3*	*FLT4*^†^	*KLB*^†^	*MS4A1*^†^	*PARP1*^†^	*PRKCQ*^†^	*RPA4*	*TEK*	*ZRSR2*^†^
BRCA2^*†^	CHEK2^*†^	*ERCC4*	*FOLR1*^†^	*KMT2A*^†^	MSH2^*†^	*PARP2*	*PRKCZ*^†^	*RPN1*^†^	TERT^*†^	
*BRD4*^†^	*CIC*^†^	*ERCC5*	*FOXL2*^†^	*KMT2C*	*MSH3*	*PAX3*^†^	*PRKDC*	*RPTOR*	*TET2*^†^	
BRIP1^*†^	*CREBBP*^†^	*ERCC6*	*FOXO1*^†^	*KMT2D*	MSH6^*†^	*PBRM1*	*PSCA*^†^	RTEL1^*^	*TFE3*^†^	
*BTK*^†^	*CRKL*^†^	*ESR1*^†^	*FRK*	*KRAS*^†^	*MSLN*^†^	*PCNA*	PTCH1^*†^	RUNX1^*†^	*TGFBR1*^†^	
*C11orf30*	*CRLF2*^†^	*ESR2*^†^	*FUS*^†^	*LAG3*^†^	*MST1R*	*PDCD1*^†^	PTEN^*†^	*RUNX1T1*^†^	*TGFBR2*^†^	
*CALR*^†^	*CRTC1*^†^	ETV6^*†^	*FYN*^†^	*LIG3*	*MTOR*^†^	*PDCD1LG2*^†^	*PTK2*^†^	*RYR1*	TMEM127^*^	

^*^Refers genes that may be reported as pathogenic or likely pathogenic incidental germline variants. ^†^Refers 284 fusion genes.

### Analytic sensitivity (PPA) and analytic specificity (PPV)

#### SNVs and indels

In each tumor sample in our analytical validation set, we identified the set of somatic small-variant calls reported by the reference method, and meeting certain criteria: (1) the variant’s allele frequency (AF) as reported by the reference test must be >5%; and (2) the variant must not have been rejected by our tumor-normal analysis because we found significant evidence for the variant in the matched-normal sample. Across the 218 tumor samples analyzed, the final truth set from the reference method consisted of 2,986 somatic SNVs and 674 somatic indels across the exome, covering 15 different tumor types. We evaluated our analytical sensitivity, and specificity, or positive predictive value (PPV), by comparing the somatic small variants reported by the NeXT Dx test to this truth set from the reference method. For SNVs, we report a positive percent agreement (PPA) of 99.4% and a PPV of 99.7% (see Table 5A). For indels, we report a PPA of 98.2% and a PPV of 99.9% (see Table 5B).

**Table 5A:** SNV accuracy

		Detected by reference method	
		Yes	No
Detected by NeXT Dx	Yes	2,957	10
	No	19	0

**Table 5B:** Indel accuracy

		Detected by reference method	
		Yes	No
Detected by NeXT Dx	Yes	661	1
	No	12	0

As the orthogonal reference method is tumor-only, and does not perform correction using a matched germline sample, there were a significant number of small variants reported by the reference method that were confirmed to be germline variants by NeXT Dx. Review of the data and bioinformatic analysis established that approximately one quarter of the mutations reported by the reference method were germline mutations and thus were removed from analysis.

### Copy number alterations (CNAs)

The performance of NeXT Dx in measuring CNAs (whole gene deletions and amplifications) was evaluated on a total of 198 samples. NeXT Dx detected 100 CNAs in 54 samples across 13 tumor types. The alterations consisted of 74 amplifications and 26 deletions. Only amplifications with a copy number greater than or equal to our predefined cutoff of 8 were called. Copy number amplifications ranged from 8 to 57 copies.

**Figure 1:**
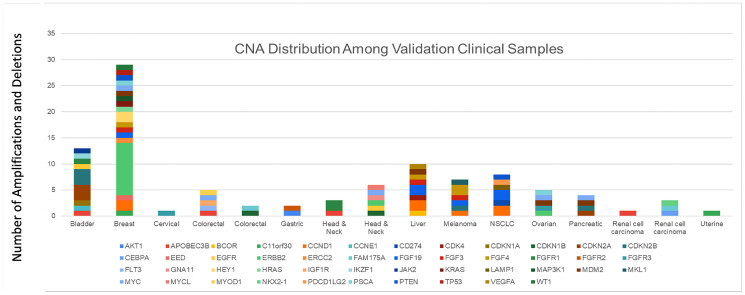
CNA distribution among validation clinical samples. A description of the CNA distribution in the validation is shown by tumor type, which represents a cross section of clinically relevant copy number alterations that would be seen in clinical samples.

The reference methods consisted of (a) panel-based NGS for amplifications in 59 overlapping genes, and (b) polymerase chain reaction (PCR) for CNAs not reported by the NGS reference method, and for whole gene deletions. A positive amplification or deletion call was based on concordance with the respective reference method. Based on the 100 alterations, the PPA between NeXT Dx and the reference methods was 98.0%, and the PPV was 100.0% (Table 5C). A description of the CNA distribution in the validation is shown by tumor type in Figure 1, which represents a cross section of clinically relevant copy number alterations that would be seen in clinical samples.

**Table 5C:** CNA accuracy

		Detected by Reference Method	
		Yes	No
Detected by NeXT Dx	Yes	98	0
	No	2	0

### Microsatellite instability (MSI)

MSI is a property of some tumors in which mutations have introduced a deficiency in DNA mismatch repair. In such tumors, the length of homopolymer and extended tandem repeat regions can diverge from the original germline length, becoming either shorter or longer. In NeXT Dx, we evaluate the MSI status of a sample by comparing the distribution of motif lengths measured at each of 117 homopolymer loci across the exome, to the distribution of lengths measured in the matched normal sample. When the length distribution in the tumor is significantly different from the length distribution in the normal, that microsatellite locus is identified as unstable. The MSI status is reported as high (“MSI-H”) if more than 10% of the 117 loci are identified as unstable, and reported as stable (“MSS”) otherwise.

The reference method also reports MSI status for each tumor sample, so to evaluate the accuracy of our MSI measurements, we simply measure the concordance of our MSI classification to theirs. Out of 212 tumor samples evaluated for MSI by both tests, the two tests applied different classification to only two samples. The PPA is 92.3% and the PPV is 100% for an overall accuracy of 99.1%.

### Tumor mutation burden (TMB)

**Figure 2:**
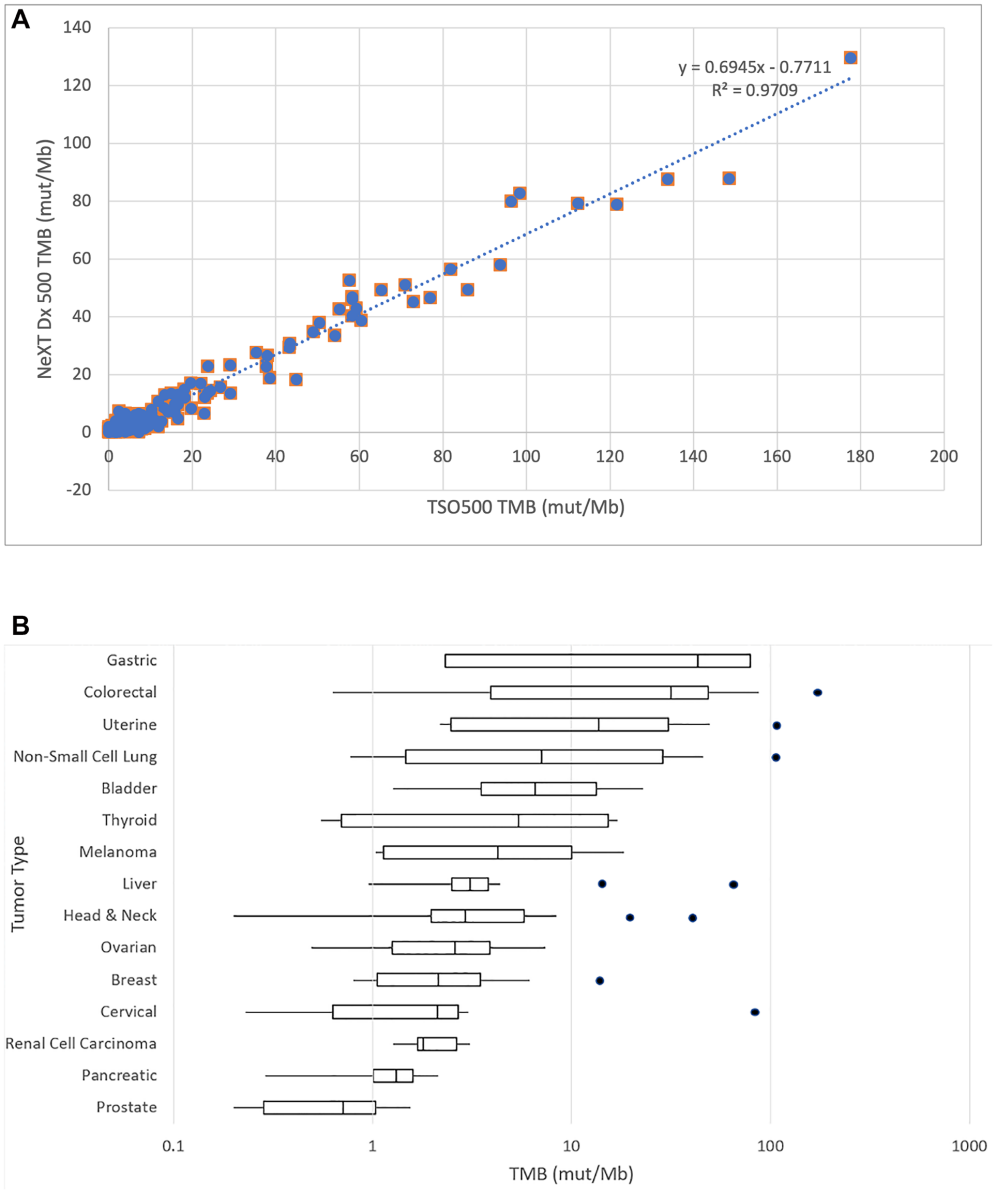
(**A**) Correlation of NeXT Dx tumor mutation burden with reference method. An XY plot of the TMB values from the two methods shows a linear regression and correlation between the methods reported in mutations per megabase. The Pearson correlation coefficient is 0.985, showing strong correlation between the methods. The NeXT Dx measures TMB by scanning the whole exome for mutations, then eliminating those that are present in the corresponding normal sample so that only tumor-associated mutations are considered. This may explain the regression slope of 0.69, as the reference method does not correct for germline mutations and thus could over-report TMB. (**B**) TMB distribution of validation samples by cancer type. The box and whiskers plot demonstrate the distribution of TMB in mutations per megabase. The TMB axis is on a logarithmic scale. Boxes denote the range from first to third quartile of TMB scores. The vertical line in each box denotes the median TMB value. The low and high whiskers indicate the lowest and highest TMB values that are within ± 1.5 times the interquartile range. Outliers outside 1.5 times the interquartile range are shown as individual points. The tumor types are ranked by median TMB from highest to lowest.

TMB is defined as the rate of occurrence of nonsynonymous somatic mutations, typically reported in units of mutations per million base pairs (mut/Mb). Because the reference method evaluates TMB based on a limited gene footprint and without the benefit of a matched normal, it is difficult to adopt their TMB values as a ground truth in this context. Nevertheless, we are able to demonstrate in Figure 2A, that the TMB values reported by the reference method and by NeXT Dx across 218 samples are highly correlated (linear regression r^2^ = 0.971, Pearson correlation coefficient = 0.985). The slope of the best-fit line is 0.694, consistent with the idea that the TMB values of the tumor-only reference method are likely overestimated.

The TMB as measured by NeXT Dx and by cancer type is illustrated in Figure 2B. The box and whiskers plot demonstrate the distribution of TMB in mutations per megabase. The TMB axis is on a logarithmic scale. Boxes denote the range from first to third quartile of TMB scores. The vertical line in each box denotes the median TMB value. The low and high whiskers indicate the lowest and highest TMB values that are within ± 1.5 times the interquartile range. Outliers outside 1.5 times the interquartile range are shown as individual points. The tumor types are ranked by median TMB from highest to lowest.

### Gene fusions

**Figure 3:**
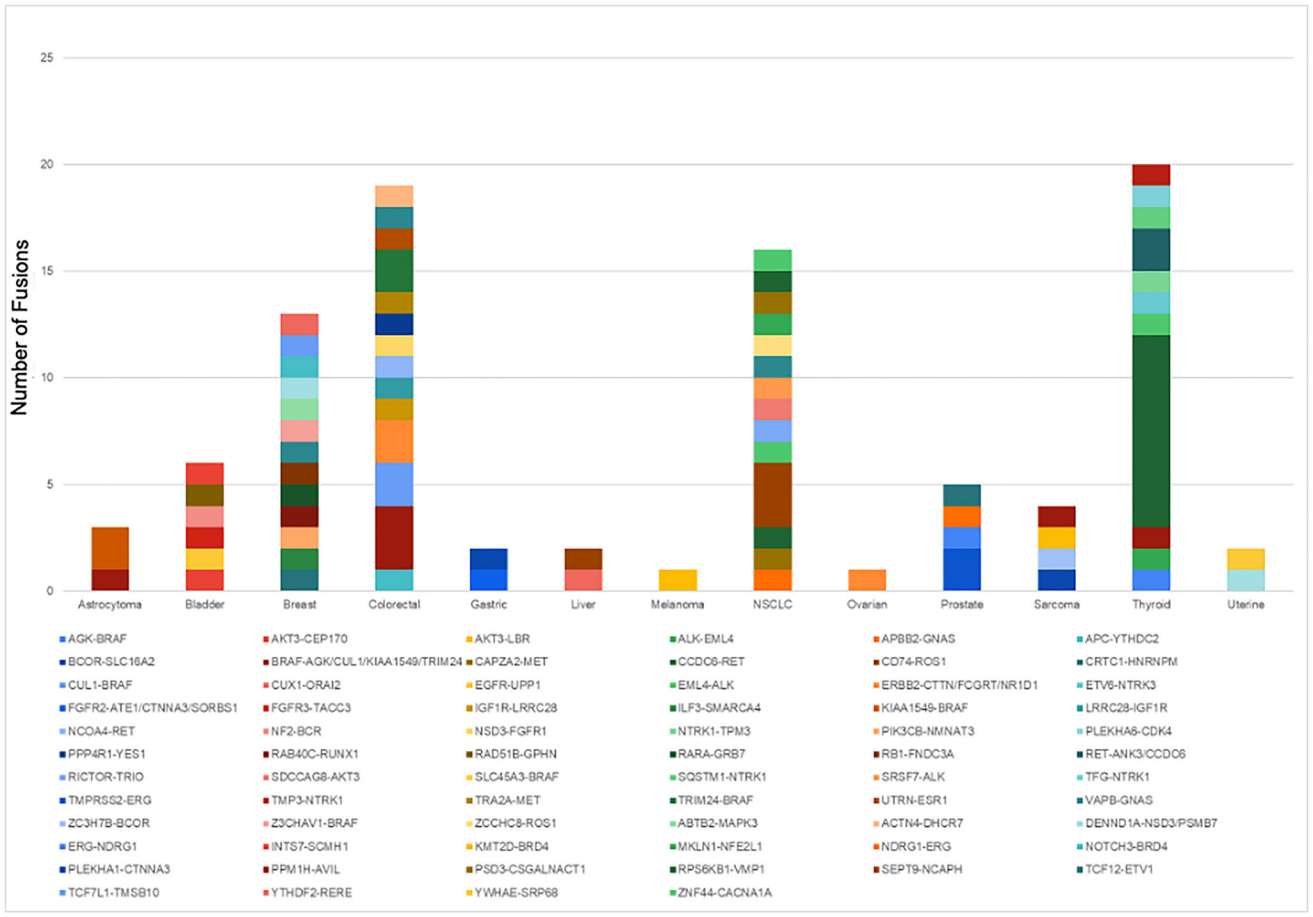
Fusions detected by NeXT Dx in the validation set. A total of 121 fusions, representing 13 different tumor types, were detected in the validation set.

Gene fusions are detected using RNA extracted from test samples. The whole transcriptome of each tumor sample is sequenced to 200 million reads. A total of 246 tumor tissue, cell line, and reference samples were tested. Out of these, 121 fusions were detected in 66 samples, across 12 different tumor types. NeXT Dx reports on 284 fusion genes, while the reference method has a 56 gene fusion footprint, so the validation utilized a second reference method, reverse transcription followed by PCR amplification and Sanger sequencing. A graph of the fusions detected in the validation set, per tumor type, is shown in Figure 3. Based on the results, NeXT Dx has a PPA of 95.8% and a PPV of 98.3% (see Table 5D).

**Table 5D:** Fusion accuracy

		Detected by Reference Method	
		Yes	No
Detected by NeXT Dx	Yes	114	2
	No	5	0

### Precision

To determine assay precision, 10 samples were evaluated for precision in the calling of SNVs, indels, CNAs, fusions, MSI and TMB. Repeatability between intra-run aliquots and reproducibility of inter-run aliquots were evaluated and compared across two different sequencing machines and across multiple days by multiple operators.

### Small variants (SNVs/indels), CNAs, and fusions - precision

Assay precision on small variants and copy number alterations was assessed by running 10 tumor-normal paired DNA samples. To determine repeatability, samples were run in triplicate and concordance among the three runs was determined through pairwise comparisons. The overall repeatability was taken as the average percent agreement across all pairwise comparisons. Repeatability results of small variants and CNAs are summarized in Table 6A.

**Table 6A:** Precision of small variants, fusions, and copy number alterations

	Repeatability (same operator, within day) overall concordance (%)	Reproducibility (inter-operator) overall concordance (%)
Small variants	98.1%	97.8%
Fusions	97.2%	96.5%
Copy number alterations	97.7%	97.0%

Reproducibility was determined by comparing runs performed by two different operators and on different days. Ten samples were run in triplicate and concordance among the three runs determined through pairwise comparison. The overall repeatability was taken as the average percent agreement across all pairwise comparisons. The results are shown in Table 6A.

Fusion repeatability and reproducibility was determined as above, except that 10 RNA samples from tumor specimens were run and concordance of gene fusion detection was measured. The results are shown in Table 6A.

### MSI precision

Four MSI-high and six MSI-stable samples were run, 10 replicates per sample, by two different operators and on different days. MSI status was called consistently across all replicates, for each of the 10 samples. Therefore, the overall precision for MSI was 100%.

### TMB precision

For TMB precision, 10 samples were run by two different operators. For the purposes of this analysis TMB ≥10 muts/Mb is considered high, TMB <10 muts/Mb is considered low. The TMB values of the 10 samples ranged roughly from three to 50 with seven high samples and three low samples.

For repeatability, each sample was run 10 times and the coefficient of variation (CV = standard deviation divided by the mean) of the TMB results was calculated. The CVs of the 10 runs ranged from 0.44 to 7.75%. The average CVs for the high and low TMB samples, as well as for all 10 samples, are shown in Table 6B.

**Table 6B:** TMB repeatability and reproducibility

	Repeatability | (single operator, between run) (CV, %)	Reproducibility (between operator) (CV, %)
TMB-High (*n* = 7)	1.01%	1.52%
TMB-Low (*n* = 3)	4.87%	6.01%
All samples (*n* = 10)	2.17%	2.87%

Reproducibility was obtained by calculating the coefficient of variation of the 10 TMB results for each sample reported by the two operators. CVs ranged from 0.60 to 8.30% across the 10 samples. In Table 6B the average of the CVs is reported for the high TMB samples, the low TMB samples, and for all samples combined. It should be noted that the cutoff threshold of TMB 10 is an arbitrary value assigned for separating low and high TMB only for comparison of precision at the low and high ranges. NeXT Dx reports TMB values as mutations per megabase only.

### Allele frequency reproducibility between operators

**Figure 4:**
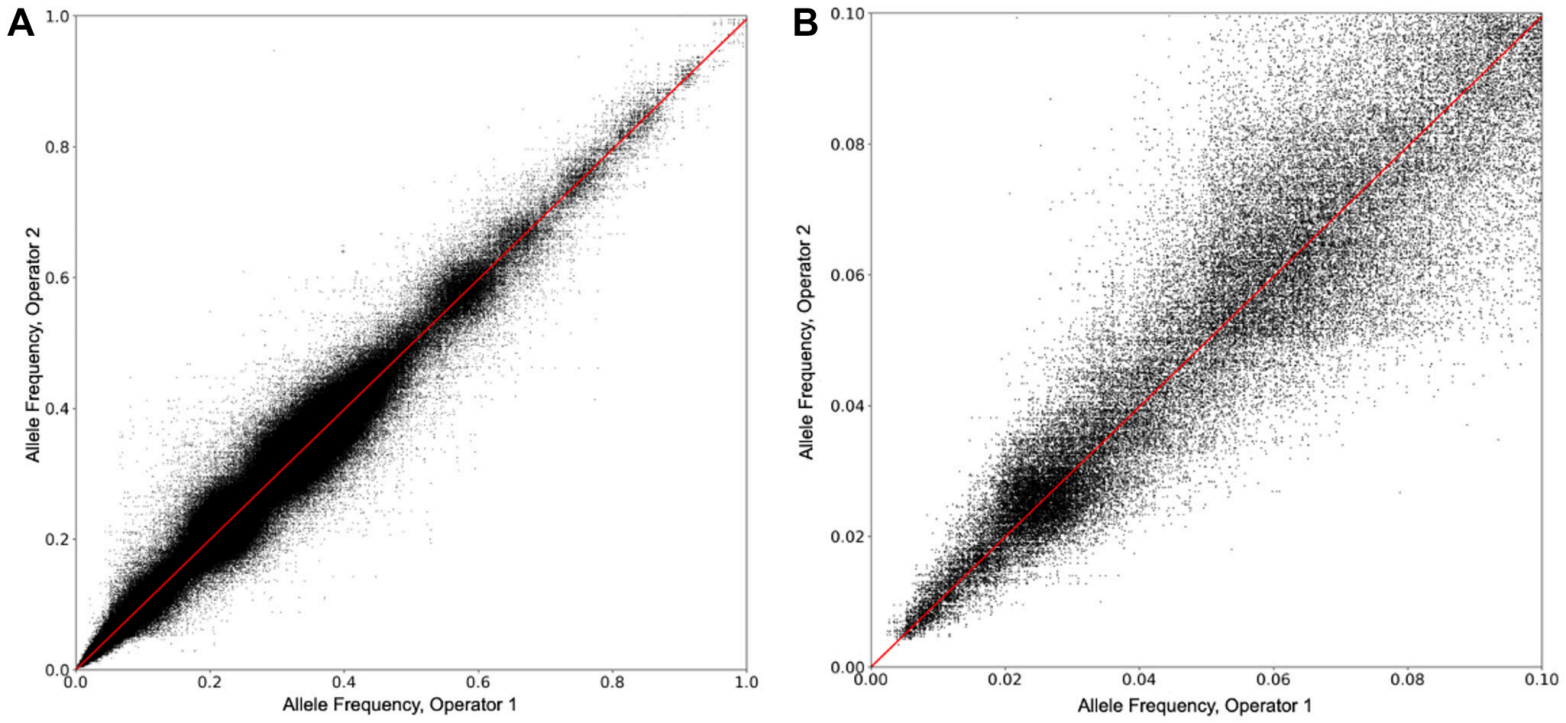
Allele frequency reproducibility between operators. (**A**) The reproducibility of AF measurements was determined between two different operators. Ten different samples were used, containing a total of approximately 2,400 SNVs. Each sample was run multiple times on different days by the two operators. A plot of the AFs measured by the two operators on the same SNV is shown. In all, 557,391 comparisons are plotted. (**B**) The data in 4a is shown, but over the AF range of 0 to 10%.

The reproducibility of allele frequency (AF) measurements was determined between two different operators. Ten different samples were used, containing a total of approximately 2,400 SNVs. Each sample was run multiple times on different days by the two operators. A plot of the concordance of results between the operators, as a function of AF, is shown in Figure 4A. In all, 557,391 comparisons are plotted. Figure 4B shows the concordance between operators in the AF range of 0 to 10%. For both figures, the correlation between operators is strong, with a regression equation of AF (Operator 2) = AF (Operator 1) × 0.995, r^2^ = 0.956.

### Limit of detection

The limit of detection (LOD) is the lowest variant AF at which the NeXT Dx measures small variants reliably. In this study reliability is considered a call rate with a PPA greater than 95%.

To determine the LOD, tumor samples were diluted with the corresponding patient normal samples to achieve dilutions of 80%, 50%, 30%, 20%, 10%, 5%, and 2.5%. Dilutions were performed on five tumor samples at two different DNA input amounts: 200 ng and 150 ng.

**Figure 5:**
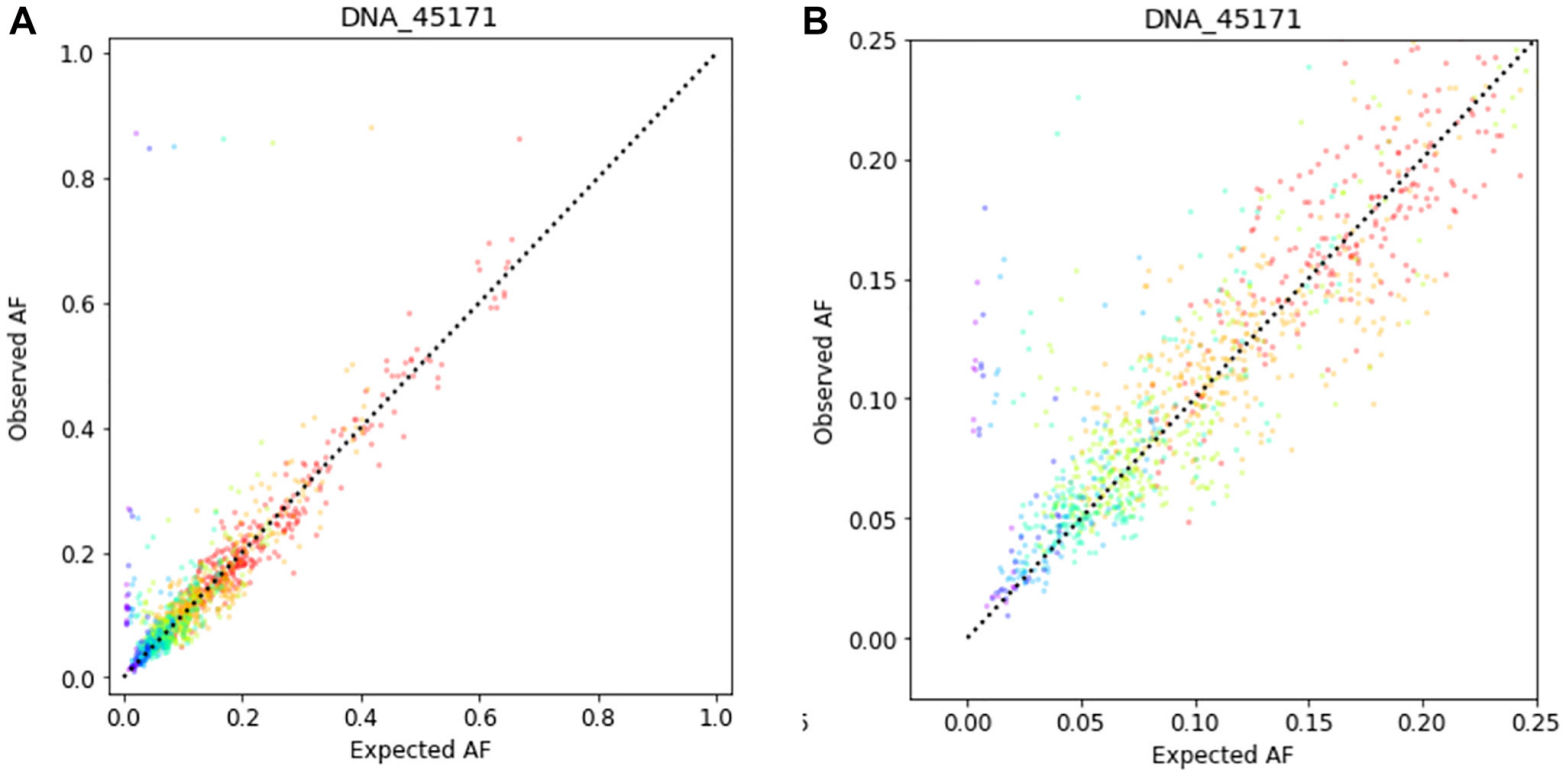
Correlation between observed AF and expected AF for 401 cancer-associated genes in a tumor tissue sample diluted with the corresponding normal specimen. The sample shown is representative of those tested in the limit of detection study. The input DNA quantity was 100 ng. (**A**) Data shown over the range 0 to 100% AF. (**B**) Data shown over the range 0 to 25% AF.

Starting with the somatic variants detected in the undiluted samples as a truth set, we compute the “expected AF” for each truth-set variant, in each dilution sample, by multiplying the measured undiluted AF by the dilution fraction. The expected AFs are well-correlated with the measured Afs in the dilution samples (Figure 5).

**Figure 6:**
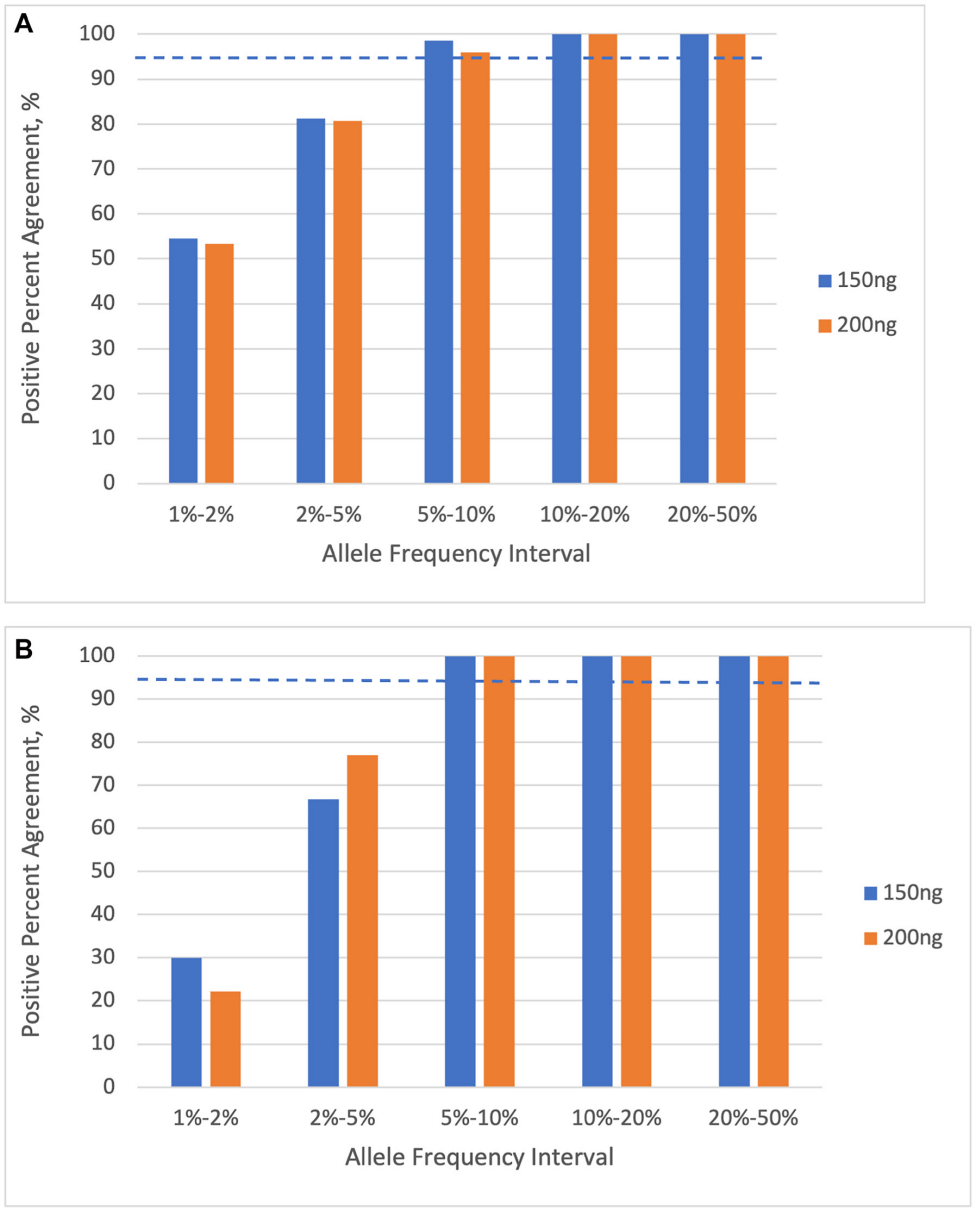
Percent positive agreement of variant calls as a function of allele frequency interval, for 401 cancer-associated genes. (**A**) single nucleotide variants (SNVs). (**B**) insertions and deletions (indels). The dashed lines indicate the 95% PPA threshold. Results are shown for input DNA quantities of 150 ng and 200 ng.

We then examined the fraction of the truth-set variants that were detected in the diluted samples, as a function of their expected AF, for variants in the reportable set of 401 cancer-associated genes (Figure 6). The LOD experiment shows a PPA above 95% in the 5–10% expected-AF bin, for both SNVs and indels. The results are very similar at both 150 ng and 200 ng inputs. This indicates that the limit of detection for SNVs and indels is between 5% and 10% AF.

## DISCUSSION

Increasingly, clinicians who are managing the care of patients with either newly diagnosed advanced cancer (typically stage III or IV), or cancer that has recurred or relapsed, or is refractory to treatment, prefer to use a comprehensive genomic profiling (CGP) test that provides a full range of information about the tumor and informs their treatment and management decisions [26, 27]. Multiple guidelines from professional organizations recommend this type of comprehensive testing. For example, the recent ASCO Provisional Clinical Opinion recommends multigene panel testing, incorporating matched tumor and normal samples and utilizing RNA for fusion detection, for these types of advanced solid tumor cases [24]. The goal of this comprehensive testing is to identify the right treatment for each patient at the right time, thereby improving survival and avoiding potentially wasteful or even harmful interventions that are not appropriate. Unfortunately, CGP tests are underutilized in community oncology practice settings, resulting in the majority of patients not being offered the most effective testing options to manage their cancer [5].

Advances in CGP technologies have led to diagnostic testing that is more sensitive, accurate, and comprehensive in the detection of genomic biomarkers for cancer therapy. For example, the composite biomarkers MSI and TMB, both detected through genomic assays, are indications for eligibility for immune checkpoint inhibitor (ICI) therapy in patients with solid tumors [28]. As well, multiple genomic markers such as gene fusions, small variants, and copy number alterations, have indicated specific approved treatments or eligibility for clinical studies.

NeXT Dx incorporates a range of features and comprehensive genome variant detection methods that lead to improved disease management and possible enhanced clinical utility. The assay utilizes the sequences of both tumor tissue and a matched normal specimen, enabling the removal of germline mutations from detected somatic mutations, leading to more accurate reporting of true somatic variants. In tumor-only sequencing, removal of germline mutations is achieved through established germline databases [24], which could result in residual false somatic mutations, especially in patients of non-European ancestry since the established germline databases are mainly derived from European populations [18]. Importantly, tumor-only sequencing also cannot distinguish the germline or somatic origin of a variant and therefore cannot report pathogenic or likely pathogenic germline variants that could be actionable. In a study of over 17,000 tumor-only test results it was found that 8.7% of the pathogenic tumor variants were actually of germline origin [29]. Next Dx reports pathogenic and likely pathogenic germline variants in 59 cancer-associated genes as incidental findings from the patient’s normal sample.

Additional important attributes of the NeXT Dx test reside in its sequencing coverage methodology. The proprietary ACE technology allows more uniform coverage of the genome, especially in GC-rich regions that other sequencing platforms may miss. When sequencing coverage of individual clinically relevant genes is compared between ACE and other commercially available exome products, there can be gaps or lower coverage (<500×) at key clinically relevant alterations such as EGFR T790M or most of the top homologous recombination repair (HRR) genes (unpublished observations). The clinical impact of these differences is actively being tested to determine if more patients could benefit from this advanced exome ACE technology. At a median sequencing depth of 500× across the exome, and a sequencing depth of >1500× in 247 important cancer-associated genes, NeXT Dx potentially provides more accurate and sensitive variant detection than other comprehensive platforms. Importantly, NeXT Dx is a WES/WTS assay, covering over 20,000 genes, allowing expansion of clinical reporting as molecular alterations with clinical significance are discovered. This is in contrast to panel-based tests that would require expanding the gene footprint of the assay and validation of new clinically relevant genes, in order to provide reporting of novel alterations tied to FDA approved drugs.

Remarkably, our validation data indicated that 33.9% of the small variants (SNVs and indels) were discordant between NeXT Dx and the reference method used in the accuracy studies, which was a limited gene panel-based tumor-only method. This illustrates a limitation of tumor-only testing, which could impact therapy selection for a cancer patient. After eliminating germline variants reported as tumor variants from the reference method data, the concordance (PPA) rate between the two methods was found to be 99.4% and 98.2% for SNVs and indels, respectively.

High TMB has emerged as an indicator for immune checkpoint inhibitor therapy response across tumors [28]. As there are a range of different assays for TMB assessment spanning a wide variety of tests, the Friends of Cancer Research (FOCR) has recently formed a consortium to attempt to harmonize these methods [30]. In Phase 2 of the FOCR harmonization initiative, 29 tumor tissue and cell line samples were distributed to 16 laboratories for determination of TMB using panel-based technologies. The results were then compared to the FOCR standard of WES-derived, tumor-normal TMB [30]. It was found that most laboratories overestimated TMB compared to the FOCR standard, and that this overestimation could be corrected to an extent by eliminating known germline variants from the TMB calculation. However, even after the corrections, TMB was still significantly overestimated, especially for samples from patients of African origin [30]. Other studies have confirmed that tumor-only derived TMB is overestimated, especially in patients of Asian or African origin [22]. This suggests that the best way to correct overestimation of TMB is through an approach where TMB is not only calculated from WES of the tumor, but is corrected for germline variants in the normal sample from the same patient. In the analytical validation study, NeXT Dx consistently reported lower TMB values compared to the tumor-only panel-based orthogonal reference test, suggesting that the potential overestimation of TMB by the comparative method was corrected through elimination of germline mutations, as was demonstrated in the FOCR study.

Gene fusions are critical and, increasingly, targetable alterations in several solid tumor types. Tests that only sequence DNA do not detect all fusions due to their gene tiling approach, which limits the detection of fusions that involve intronic regions [31, 32]. It is estimated that DNA-based methods of fusion detection will miss approximately 15% to 30% of fusions that have a potential clinical impact [33, 34]. For example, it is estimated that 64% of NTRK3 fusions will not be detected by DNA methods, while NTRK fusions generally have an 80% response rate to tyrosine kinase inhibitor therapy [23, 35]. Sequencing of the transcriptome to detect fusions in the RNA has been recognized as the preferred detection method for comprehensive fusion detection [24]. Recently, the National Comprehensive Cancer Network (NCCN) recommended that non-small cell lung cancer patients who in broad panel testing did not have identifiable driver oncogenes, should consider RNA-based NGS to maximize detection of fusion events [36]. RNA sequencing by NeXT Dx performs 200 million reads per sample while other commercially available approaches typically attain at maximum 50–100 million reads per sample. Increased sequencing depth of the RNA should lead to increased sensitivity for rare fusions, although the clinical relevance of this has not been demonstrated. Compared to many current commercially available methods which report a limited number of clinically relevant fusions, as shown in the validation data, NeXT Dx reports fusions from 284 genes. The broad 284 gene reporting footprint for fusions includes tumor suppressor genes. Traditionally, gene fusions in cancer are associated with activation, but fusions involving genes such as APC and NF2 have been shown to result in loss of protein function in the same way that a nonsense or frameshift DNA sequence variant would [37, 38]. For example, Choi et al. [38] found that in colorectal cancer (CRC) patients the APC-COMMD10 fusion led to a truncation of the APC gene, likely resulting in loss of tumor suppressor function in the APC protein. The NeXT Dx report includes these important variants.

MSI occurs from defects in the mismatch repair system and can be measured from expanded nucleotide repeats in microsatellite loci throughout the genome. Importantly, high MSI (MSI-H) is an indicator for ICI therapy across solid tumors for patients with unresectable or metastatic cancer who have progressed on prior treatment and have no satisfactory treatment options [28]. Initially MSI-H was diagnosed if extended short tandem repeats occurred in two or more of the five loci of the National Cancer Institute (NCI) panel, BAT25, BAT26, D2S123, D5S346 and D17S250 [39]. In more recent years the panel has been refined [40, 41], and with the advent of NGS [41], some laboratories have extended the panel to multiple loci, with the objective of assessing MSI in a range of tumors, not just colorectal cancer as was the main intent of the original NCI panel [41, 42].

NeXT Dx involves sequencing a matched normal sample in all cases, with the aim of providing a more accurate report of somatic variants in the tumor. In addition, NeXT Dx identifies pathogenic or likely pathogenic variants in the germline from 59 genes and reports those as incidental findings with a recommendation that they should be followed up by genetic counseling and confirmatory testing. In the validation sample set, NeXT Dx identified pathogenic BRCA2 germline variants in colorectal and head and neck cancer cases; these tumor types would typically not be referred for germline testing in the absence of other personal and family history risk factors (per guidelines), and tumor-only assays are unable to establish the origin of detected variants. Further, patients with tumor types and/or family histories deemed to be at increased risk for hereditary origination do not receive recommended germline testing as frequently as they should [43–46]. A tumor-normal test report such as that provided with NeXT Dx testing may identify pathogenic or likely pathogenic variants and indicate the need for germline follow up testing in any solid tumor type, thus allowing even patients without significant hereditary risk factors to be potentially eligible for germline testing.

Further clarifying the differential value of tumor-normal testing over tumor-only testing, in this study a total of 1,246 germline small variants out of approximately 4,900 small variants were reported as tumor variants by the reference method. For a treating clinician, these additional, reported variants complicate clinical interpretation and may lead to inappropriate patient management decisions.

In this analytical validation study, NeXT Dx has been shown to accurately detect SNVs, indels, CNAs, and gene fusions, and determine MSI and TMB. We took an agnostic approach to validating the exome by combining analysis of over 3800 alterations in over 200 clinical samples, allowing us to have the power to calculate analytic sensitivities with tight confidence intervals and thus highly accurate results. The assay sensitivity has been demonstrated for SNVs and indels to an allele fraction level of >5%. Both within-run repeatability and between-day reproducibility of all measured parameters showed agreement to the extent of 92.1% or better. As TMB is reported as a numerical value, its precision was shown to have an average coefficient of variation of 1.01 to 1.52% for high values (TMB ≥10 mut/Mb), and an average coefficient of variation of 4.87 to 6.01% at low values (TMB <10 mut/Mb).

Accuracy across all variant measurements, as determined by comparison with reference methods, ranged from 95.8% to 100.0%. In the case of TMB, numerical correlation with the reference method yielded a Pearson correlation coefficient of 0.985.

Tumor-normal assays, such as NeXT Dx, offer important improvements in the accuracy of variant detection that is of somatic origin, and are therefore preferable to tumor-only assays [47–49]. Fusion detection via RNA analysis is the preferred method to identify this important class of variants, which increasingly play a “tumor agnostic” role in treatment decision- making [23, 31–35]. Additionally, the calculation of composite biomarkers used for therapy selection, such as TMB, is more accurate when performed in a tumor-normal context, especially for patients from minority and/or underrepresented populations [22].

Cancer has been increasingly recognized as highly heterogeneous, with differences in the genetics of each patient’s tumor potentially driving differential treatment even within the same tumor type. By more comprehensively characterizing the molecular characteristics of each patient’s tumor, NeXT Dx provides personalized recommendations critical to clinical decision-making with respect to current FDA-approved drug-variant specific treatments and evolving treatment opportunities via enrollment in clinical trials.

## MATERIALS AND METHODS

### Reference materials

The validation study was performed with patient tumor tissue samples and matched normal specimens obtained from blood or proximal normal tissue, except where cell lines and reference materials were used.

The following reference materials were used in specific sections of the study.

SeraCare Seraseq FFPE Tumor Fusion RNA reference material (Material Number 0710-0496, SeraCare, Milford, MA) was used in the gene fusion validation.

MDA-MB-175 cell line (containing an NRG1 fusion variant, (AMSBIO, Cambridge, MA, USA) was used in the gene fusion validation study.

HCC1187 cell line (ATCC) was used in the limit of detection study.

HCC1395 cell line (ATCC) was used in the limit of detection study.

NCI-H2126 cell line (ATCC) was used in the limit of detection study.

### Sample processing and nucleic acid extraction

For FFPE tissue specimens, a pathologist used standard H&E staining to evaluate tumor content. When necessary, macrodissection was performed to bring the neoplastic content to 30% or above. DNA and RNA were extracted from FFPE using the Qiagen AllPrep DNA/RNA FFPE Tissue Kit (Qiagen, Germantown, MD). DNA and RNA were extracted from fresh frozen tissue using the Qiagen AllPrep DNA/RNA Mini Kit (Qiagen). Genomic DNA was extracted from peripheral blood using the QIAsymphony DSP DNA Mini Kit with QIAsymphony automation (Qiagen). Quality control metrics established from prior studies were used to evaluate DNA and RNA quality.

### DNA/RNA quality control

Nucleic acid quality and quantity were checked at various stages throughout the laboratory process. After extraction, DNA and RNA were tested for quality by measuring the DNA Integrity Number (DIN) and RNA Integrity Number (RIN), respectively, on the Agilent TapeStation (Agilent Technologies, Santa Clara, CA). RNA was also tested for DV200 (Agilent Technologies). DV200, a measure of the percentage of RNA fragments > 200 nucleotides, was used because mean RNA fragment size is a more reliable determinant of RNA quality for RNA library preparation. Qualified RNA samples must have a DV200>30%. Nucleic acid concentration was determined using Qubit fluorometry. Final QC of DNA prior to enrichment was performed with A260/A230 spectrophotometry and SYBR-green quantitative PCR.

### DNA and RNA library construction and sequencing

Construction of DNA and RNA based libraries was performed using standard molecular biology techniques and proprietary sets of primers. KAPA HyperPrep Kits (Roche) were used for DNA library preparation and KAPA Stranded RNA-Seq kits (Roche, Indianapolis, IN) were used for RNA library preparation.

In brief, 150 ng to 750 ng of DNA for each tumor and normal sample was mechanically sheared to an average size of 225 base pairs (bp) using a Covaris ultrasonicator. After ligation to Personalis primers, libraries were amplified, captured using the NeXT Dx probe set, and re-amplified. For RNA, 50 to 200 ng of RNA for each tumor sample was heat fragmented in the presence of magnesium to a suitable size profile for library creation. RNA was converted to DNA using a randomly primed reverse transcription reaction followed by second strand synthesis. After ligation to Personalis primers, libraries were amplified, captured using the NeXT Dx probe set, and re-amplified.

Amplified captured libraries were sequenced on an Illumina NovaSeq6000 to a minimum of 30 gigabases for RNA based tumor libraries, 135 gigabases tumor derived DNA based libraries, and 25 gigabases for normal derived DNA based libraries. Sequencing data was compared to internal QC standards prior to progressing to analysis.

### Analysis pipeline

FASTQ files from the tumor and normal DNA samples were initially processed through the Personalis Core DNA analysis pipeline, where standard secondary analyses are performed in a best-practices GATK workflow [50]. Reads are aligned to the GRCh37 human reference assembly using bwa-mem. Duplicate reads are removed, and reads in the vicinity of candidate indel variants are realigned. Finally, the base quality scores are recalibrated. In addition to the standard read-alignment workflow, additional analyses are performed including HLA typing, measuring QC metrics, and germline variant calling.

The aligned reads from the Tumor and Normal DNA samples are then passed into the Cancer DNA Somatic analysis pipeline, where the tumor and normal reads are co-analyzed to identify tumor-specific somatic small variants. The Mutect and Vardict pipeline software tools are analyzed to call somatic SNVs and Indels, and apply proprietary filtering to determine a final high-quality call set. In addition to somatic small-variant calling, the somatic pipeline also uses an internal algorithm to call somatic CNA events, and to measure the MSI status of the tumor sample.

FASTQ files from the tumor RNA sample are processed through the Cancer RNA analysis pipeline. Ribosomal reads are identified and removed from the FASTQs by aligning against a reference of ribosomal contigs. Non-ribosomal RNA reads are aligned to the GRCh37 human reference assembly using a splice-junction-aware RNA aligner (STAR [51]). The STAR alignments are used to measure gene expression across the exome, as well as transcript-specific expression. In addition, the pipeline utilizes a third-party tool, Arriba [52], to call somatic fusion events from the STAR-aligned reads.

## Abbreviations

ACE: Accuracy and Content Enhanced
ACMG: American College of Medical Genetics
ASCO: American Society of Clinical Oncology
ATCC: American Type Culture Collection
Bp: base pairs (bp)
CLIA: Clinical Laboratory Improvement Amendments
CAP: College of American Pathologists
CRC: colorectal cancer
CGP: Comprehensive genomic profiling
CNAs: Copy number alterations
FFPE: Formalin fixed paraffin embedded
FOCR: Friends of Cancer Research
ICI: Immune checkpoint inhibitor
Indels: Insertions and deletions
LDT: Laboratory developed test
MSI: Microsatellite instability
NCCN: National Comprehensive Cancer Network
NGS: Next generation sequencing
NSCLC: Non-small cell lung cancer
PCR: Polymerase chain reaction
PPA: Positive percent agreement
PPV: Positive predictive value
SNP: Single nucleotide polymorphism
SNV: Single nucleotide variants
TMB: Tumor mutation burden
WES: Whole Exome Sequencing
WTS: Whole Transcriptome Sequencing
